# Plasmablast Expansion Following the Tetravalent, Live-Attenuated Dengue Vaccine Butantan-DV in DENV-Naïve and DENV-Exposed Individuals in a Brazilian Cohort

**DOI:** 10.3389/fimmu.2022.908398

**Published:** 2022-06-28

**Authors:** Cássia G. T. Silveira, Diogo M. Magnani, Priscilla R. Costa, Vivian I. Avelino-Silva, Michael J. Ricciardi, Maria do Carmo S. T. Timenetsky, Raphaella Goulart, Carolina A. Correia, Mariana P. Marmorato, Lilian Ferrari, Zelinda B. Nakagawa, Claudia Tomiyama, Helena Tomiyama, Jorge Kalil, Ricardo Palacios, Alexander R. Precioso, David I. Watkins, Esper G. Kallás

**Affiliations:** ^1^ Division of Clinical Immunology and Allergy, School of Medicine, University of São Paulo, São Paulo, Brazil; ^2^ Department of Pathology, University of Miami Miller School of Medicine, Miami, FL, United States; ^3^ Department of Infectious and Parasitic Diseases, School of Medicine, University of São Paulo, São Paulo, Brazil; ^4^ Virology Centre, Instituto Adolfo Lutz, São Paulo, Brazil; ^5^ Division of Clinical Trials and Pharmacovigilance, Instituto Butantan, São Paulo, Brazil; ^6^ Pediatrics Department, School of Medicine, University of São Paulo, São Paulo, Brazil

**Keywords:** dengue, dengue infection, dengue vaccine, plasmablast, humoral response

## Abstract

An effective vaccine against the dengue virus (DENV) should induce a balanced, long-lasting antibody (Ab) response against all four viral serotypes. The burst of plasmablasts in the peripheral blood after vaccination may reflect enriched vaccine-specific Ab secreting cells. Here we characterize the acute plasmablast responses from naïve and DENV-exposed individuals following immunization with the live attenuated tetravalent (LAT) Butantan DENV vaccine (Butantan-DV). The frequency of circulating plasmablasts was determined by flow cytometric analysis of fresh whole blood specimens collected from 40 participants enrolled in the Phase II Butantan-DV clinical trial (NCT01696422) before and after (days 6, 12, 15 and 22) vaccination. We observed a peak in the number of circulating plasmablast at day 15 after vaccination in both the DENV naïve and the DENV-exposed vaccinees. DENV-exposed vaccinees experienced a significantly higher plasmablast expansion. In the DENV-naïve vaccinees, plasmablasts persisted for approximately three weeks longer than among DENV-exposed volunteers. Our findings indicate that the Butantan-DV can induce plasmablast responses in both DENV-naïve and DENV-exposed individuals and demonstrate the influence of pre-existing DENV immunity on Butantan DV-induced B-cell responses.

## Introduction

Dengue is a mosquito-borne viral infection that persists as a major global health problem, with up to 100 million cases every year (1). The occurrence of dengue cases in Brazil is one of the highest reported globally; between 2008 and 2019, more than 90% of the 10,6 million cases of arboviral infections reported in the country were dengue-related, and 6,429 deaths were confirmed (2). Even though a 43% reduction in cases was seen between 2020 and 2021, probable dengue cases increased more than 30% in the beginning of 2022 compared to the same period of the previous year, and 812 dengue-related deaths were reported since 2020 (3–5).

All four dengue virus (DENV) serotypes are endemic in Brazil, triggering cyclic outbreaks every few years (6). Although most dengue cases are thought to be asymptomatic, the infection can produce a spectrum of symptoms ranging from a self-limiting but debilitating acute febrile disease to more severe and potentially life-threatening plasma leakage syndrome, dengue hemorrhagic fever or dengue shock syndrome (7). One of the best-characterized risk factors for a severe disease course is prior exposure to a different DENV serotype. Indeed, in a secondary DENV infection, immune responses generated after primary infection may paradoxically intensify disease severity as poorly neutralizing serotype-cross-reactive antibodies (Abs) facilitate virus entry into host cells, in a mechanism known as antibody-dependent enhancement (ADE) (8–10). This is particularly concerning as the prevalence of prior exposure to dengue in Brazil is high, all 4 DENV serotypes circulate in the country, vectors are widely distributed, and the transmission area has been increasing over time due to environmental changes (6). Thus, it raises the importance of studying the immune profile after vaccination in an exposed population.

Humoral and cellular immune responses play important roles in both DENV disease pathogenesis and protection (11). During acute DENV infection, activated naïve or memory B cells proliferate and differentiate into plasmablasts that leave the germinal centers to transiently enter the peripheral blood. This results in a peak of highly enriched antigen-specific Ab secreting cells, following a few days of infection (12, 13). High titers of neutralizing Abs (nAb) against the virus are relevant mediators of protection against subsequent DENV infection (14). Robust nAb responses developed after DENV infection are believed to provide lifelong protection against reinfection with the same DENV serotype and short-lived protection against heterologous DENV serotypes (15). Accordingly, the induction of protective levels of homotypic nAb against each DENV serotype is one of the major goals of a dengue vaccine.

After vaccination or infection, recently activated plasmablasts derived from lymphoid organs migrate towards the peripheral blood (16). This B-cell proliferation is rapidly followed by increased virus-nAb titers in the serum (17–21). The expansion of circulating plasmablasts is also observed in DENV-infected individuals 6-10 days after the onset of fever (12, 19) and correlates with an increase in DENV-specific nAb titers (19, 22). Given the associations with late Ab immune responses, it has been postulated that plasmablast expansion is one of the earliest biomarkers of immunogenicity (23).

The live-attenuated tetravalent (LAT) Butantan DENV vaccine (DV) is a lyophilised version of TV003 vaccine, produced by the Butantan Institute, and they have been shown to be analogous vaccines (24). TV003 has been shown to protect against DENV infection in humans by eliciting humoral responses that are known to play an important role in both DENV disease pathogenesis and protection (25). Indeed, a recent study demonstrated the plasmablast expansion among participants of the TV003 Phase I clinical trial, with an increased frequency of these cells 21 days after vaccination (23). Here we followed plasmablast responses after the administration of a single dose of the Butantan-DV in a cohort of DENV-naïve and DENV-exposed participants enrolled in the Phase II clinical trial (NCT01696422). We investigate whether pre-existing antibodies to DENV serotypes alter the Butantan-DV plasmablast expansion.

## Materials and Methods

### Ethics Statement

Clinical data and blood samples analyzed in this study were obtained from the first 40 participants from the São Paulo cohort, in Brazil, enrolled in the Phase II randomized, double-blind, placebo-controlled trial of Butantan-DV (ClinicalTrials.gov identifier NCT01696422) (24) performed at the School of Medicine and the Children’s Institute – Hospital das Clínicas, University of São Paulo. The study design and protocol were approved by the local Institutional Review Board (CAPPesq, Research Projects Ethics Committee, protocol #55308) and the National Commission for Research Ethics (CONEP, protocol #155.880). All study procedures were approved by CAPPesq (protocol #1.213.202) and written informed consent was obtained from all study volunteers.

### Human Participants, Vaccine and Blood Samples

Healthy male and nonpregnant female volunteers aged between 18 and 59 years old with unremarkable findings in physical examination, blood counts, serum chemistry, and negative serology for hepatitis B, hepatitis C, and HIV, who were randomly assigned into two double-blinded groups at a ratio of approximately 2:1. The vaccine group received one dose of the lyophilized LAT Butantan-DV (n=27). The vaccine consisted of 1,000 PFU of each attenuated serotype virus (rDEN1Δ30, rDEN2/4Δ30(ME), rDEN3Δ30/31, and rDEN4Δ30) (26). Placebo inoculations consisted of a single dose of vehicle only (qualified Leibovitz L-15 medium).

These participants were tested for DENV exposure using plaque reduction neutralization test (PRNT) (24) at recruitment (up to six months prior to the beginning of the trial) and at baseline and they were categorized as either DENV-seronegative/naïve (vaccinees, n=13; placebos, n=4) or DENV-seropositive/pre-exposed (vaccinees, n=14; placebos, n=9) for one or more of the four DENV serotypes. The majority of DENV-seropositive volunteers had experienced asymptomatic dengue and therefore it was not possible to determine the time between primary DENV infection and enrollment in the study. Demographics, baseline DENV serostatus and rash occurrence after vaccination are presented in **Table 1**.

**Table 1:** Baseline characteristics of the 40 participants enrolled in the Phase II Butantan-DV Clinical Trial.

Characteristics	Placebon (%)	Vaccineesn (%)
Age, years in median (IQR)	51 (37.5-54)	45 (32.5-52)
Sex		
Female	8 (61.5)	22 (81.5)
Male	5 (38.5)	5 (18.5)
Prior YF vaccination reported		
Yes	8 (61.5)	7 (25.9)
No	5 (38.5)	19 (70.4)
Unknown	0	1 (3.7)
PRNT data at baseline		
DENV-naïve	4 (30.8)	13 (48.1)
DENV-exposed	9 (69.2)	14 (51.9)
1 serotype	8 (88.8)	9 (64.2)
DENV1	4	5
DENV2	2	1
DENV3	1	1
DENV4	1	2
≥ 2 serotypes	1 (11.2)	5 (35.8)
DENV2,4	0	1
DENV1,2,3	0	1
DENV1,3,4	0	1
DENV1,2,3,4	1	1

n, number of subjects; IQR, interquartile range.

**Figure 1:**
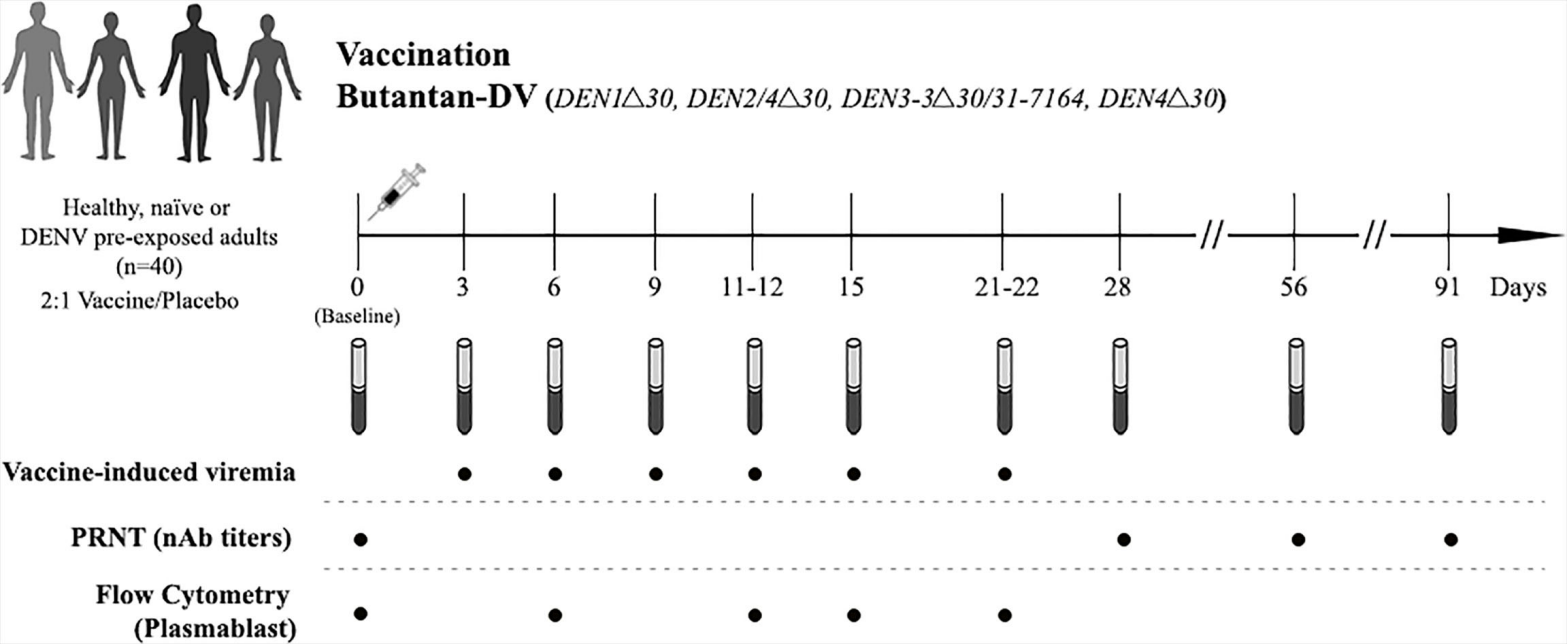
Study design. Naïve and DENV-pre-exposed volunteers received a single subcutaneous dose of the lyophilized vaccine consisting of 1,000 PFU of each attenuated virus or placebo (qualified Leibovitz L-15 medium only). Blood samples were collected pre-vaccination (baseline, day 0) and at days 3, 6, 9, 11-12, 15, 21-22, 28, 56 and 91 post-vaccination, and used for the assessment of vaccine induce-viremia, DENV neutralizing antibodies (nAbs) titers using plaque reduction test (PRNT), and plasmablast responses using flow cytometry.

All study participants were followed-up after vaccination (at days 3, 6, 9, 12, 15, 22, 28, 56 and 91) for clinical assessments, physical examination and blood collection as shown in **Figure 1**. The trial was conducted in the second half of 2014, prior to the Zika virus outbreak in this region.

### Pre- and Post-Vaccination Serostatus Analysis

The nAb response was measured by PRNT, as described previously (24) in serum samples obtained at baseline and follow-up visits (28, 56 and 91 days after vaccination). The lowest serum dilution producing ≥50% reduction in viral plaques (PRNT50) was used as the final test result in accordance with other LATV evaluations (26). PRNT50 assays used a 1:5 initial serum dilution.

Samples were tested with all four DENV serotypes. At baseline, seropositivity for each DENV serotype was defined as a nAb titer ≥1:10. Seroconversion at follow-up visits was defined as PRNT50 cut off (≥1/10) for DENV-naïve subjects and four-fold or higher increase in PRNT50 titer for DENV exposed subjects.

### Vaccine Virus Detection

The presence of vaccine viruses was assessed in all participants from 3 to 21 days after vaccine administration. Sera samples were incubated in Vero cells culture and the supernatant was analyzed through real-time RT-PCR, as previously described by Johnson and colleagues (27) and published by Kallas et al. (24). To distinguish vaccine strains from wild-type DENV, all positive samples were sequenced to identify the Δ30 deletion in the 3´ UTR of the vaccine viruses.

### Flow Cytometry

Staining for analytical flow cytometry of plasmablasts was performed as previously described (12) on fresh whole blood collected at baseline and at days 6, 12, 15 and 22 post-vaccination. Briefly, cells were labeled with the appropriately titrated Abs, followed by lysis of erythrocytes (BD FACS lysing solution, BD Biosciences). For the flow experiments, cells were resuspended in MACS buffer containing 1% formalin and ran through a FACS Canto (BD Biosciences). Flow cytometry data were analyzed using FlowJo software (TreeStar, Inc.). The percentage of plasmablasts (identified herein as CD20^-/low^ [L27, PerCP; BD Biosciences, Cat # 347674], CD27^high^ [O323, APC; Biolegend, Cat #302810] and CD38^high^ [HN7, FITC; BD Biosciences, Cat #340927] cells) was determined by using a boolean gate on the live (cell viability marker, Live/Dead, CF-594, Life Technologies) CD3^-^ (UCHT1, CF-594; BD Biosciences, Cat #562280), CD14^-^ (MφP9, CF-594; BD Biosciences, Cat #562335) and CD19^+^ (SJ25C1, APC-Cy7; BD Biosciences, Cat #557791) cell population (**Supplementary Figure 1**).

### Statistical Analysis

Continuous variables were assessed using a *t*-test or the nonparametric Wilcoxon rank-sum test when applicable. Categorical variables were evaluated with the Pearson chi or the Fisher exact test. P-values < 0.05 were considered statistically significant.

## Results

We assessed plasmablast responses in 40 individuals who were randomly assigned to receive a single dose of Butantan-DV (n=27) or placebo (n=13) in the phase II clinical trial (24). No statistically significant differences in demographic data were observed between the groups. Participants underwent blood sampling and clinical evaluations at baseline and periodically from 3 to 91 days after vaccination. As previously reported (24), rash was commonly observed following vaccination, occurring in 67% of vaccinees between days 9 and 15 post-vaccination. Detectable viremia for one or more vaccine DENV strains was found in 18 (66,7%) of 27 Butantan-DV recipients, regardless of previous DENV serostatus (**Supplementary Table 1**). It was mostly detected at day 9 post-vaccination for both DENV-naïve and pre-exposed individuals and DENV-1 was the most prevalent vaccine serotype. DENV-4 vaccine serotype was not identified in any Butantan-DV recipients.

**Figure 2:**
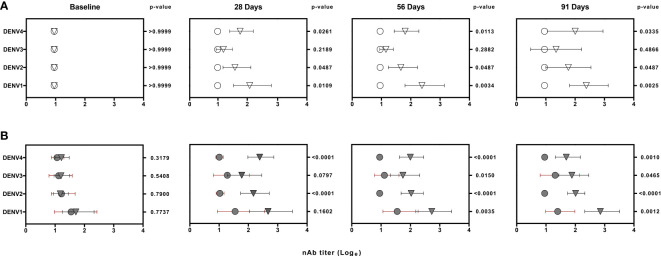
Logarithm-transformed DENV neutralizing antibody (nAb) titers from naïve (white) **(A)** and DENV pre-exposed (gray) **(B)** volunteers at baseline and post-vaccination. Data was generated by 50% plaque-reduction neutralization titer assay (PRNT50). Circles and triangles represent median log titers for each DENV serotype in placebo and vaccinees volunteers, respectively. Horizontal lines span the 25^th^-75^th^ percentiles (interquartile range). The values on the right of each time point column represents the p-values obtained after the analysis of placebo and vaccinees nAb titers using non-parametric Wilcoxon rank-sum test.

The median of nAb titers to DENVs 1 to 4 assessed before vaccination as well as at 28, 56 and 91 days following one dose of Butantan-DV are shown in **Figure 2**. Among vaccinees with confirmed primary DENV exposure, nAb titers from each DENV strain were significantly increased compared to placebo. Median log titers among DENV naïve vaccinees were also significantly higher to DENVs 1, 2 and 4 but not to DENV-3 that were not significantly increased at any post-vaccination time point evaluated. The rates of vaccine-associated seroconversion for three or more DENV serotypes in naïve and pre-exposed vaccinees were 84.61% and 92.86%, respectively. The vaccine seroconversion to DENV serotypes was detected regardless the presence of detectable viremia for the vaccine DENV strains.

**Figure 3:**
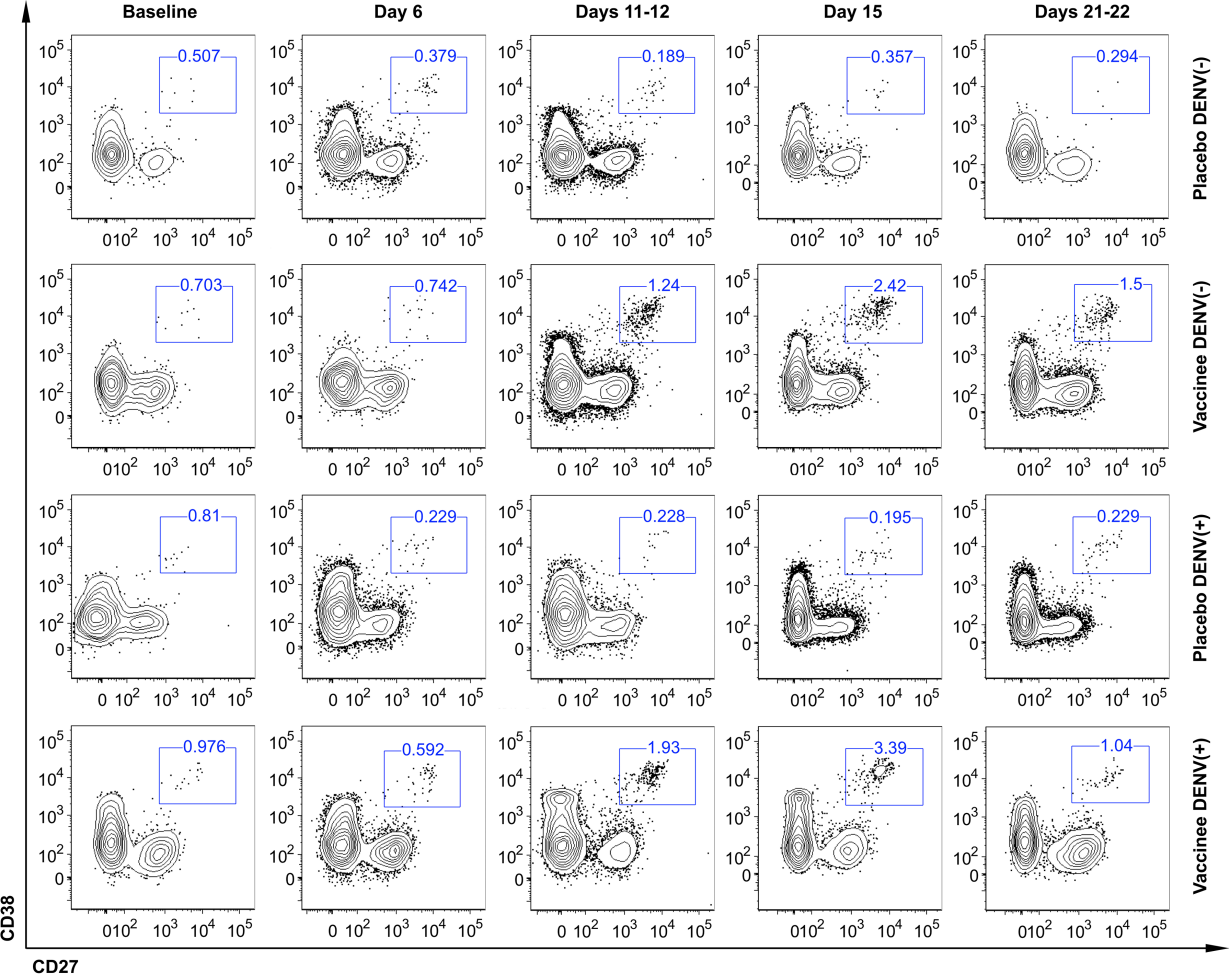
Representative flow cytometric analysis of circulating plasmablast in naïve (DENV (–)) and DENV-pre-exposed (DENV(+)) volunteers enrolled in the Phase II clinical trial evaluated before (baseline) and after vaccination (days 6, 11-12, 15, 21-22). All plots represent the frequency of CD27^+^ CD38^+^ cells gated on live CD3^-^ CD14^-^ CD19^+^ cells.

**Figure 4:**
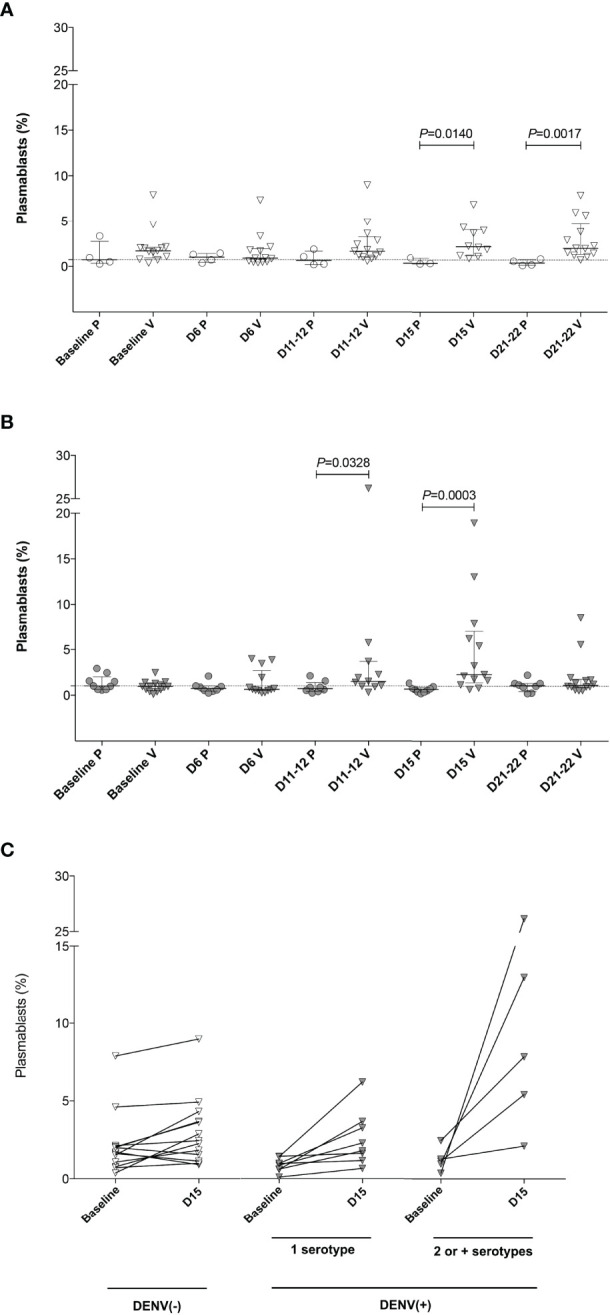
Plasmablast expansion (as the percentage of CD20^-^ CD27^+^ CD38^+^ cells among the CD19^+^ population) after Butantan-DV vaccination. Results are presented as pooled total plasmablast cells analyzed in a Boolean analysis. **(A, B)** Frequency of plasmablast in fresh blood from DENV (–) **(A)** and DENV(+) **(B)** volunteers at baseline and days 6, 11-12, 15 and 21-22 post-immunization. D, days post-vaccination. P, placebo. V, vaccinee. Horizontal bars represent median and interquartile range. Statistical analysis was performed using a non-paired t test. **(C)** Difference in the frequency of plasmablast comparing the values obtained at baseline and at the peak of plasmablast expansion (D15 after Butantan-DV vaccination) in naïve [DENV (–)] and DENV pre-exposed [DENV(+)) vaccinees. DENV(+) volunteers were divided into individuals pre-exposed to 1 or 2 or more serotypes, according to PRNT50 results to all four DENV serotypes at baseline. Data were analyzed with the Mann-Whitney U test.

To characterize the dynamics of the plasmablast response induced by the Butantan-DV, as an indicator of vaccine-induced B-cell activation, we performed flow cytometric analysis of plasmablasts in fresh blood samples collected at several time points after immunization. **Figure 3** shows representative examples of this analysis between day 0 and day 22 post-vaccination in all groups. Samples obtained at day 6 after immunization contained relatively low numbers of circulating plasmablasts, often indistinguishable from the number seen in pre-vaccination samples or collected from placebo recipients at day 6 (**Table 2**). However, samples obtained from both DENV-naïve and -exposed vaccinated participants showed an increase in plasmablast percentages at days 11-12, peaking by day 15 post-vaccination. Interestingly, plasmablast levels peaked earlier, at day 12 (**Figures 4A** **, B**) in a few individuals. Most vaccinees presented a substantial induction of circulating plasmablasts, up to 26% of the total peripheral CD19^+^ B cells in some cases. No statistically significant difference in the amount of plasmablast at peak was observed between DENV-naïve and -exposed vaccinees. In pre-exposed vaccinees we observed a similar percentage of plasmablast response regardless of the DENV serotype detected at baseline. Blood from two naïve and five pre-exposed vaccinees contained more than 3 x 10^3^ plasmablasts per ml of blood at day 15 (DENV-naïve, median 1 x 10^3^/ml; DENV-exposed, median 1.5 x 10^3^/ml). This corresponds to a two- and three-fold increase, respectively, of their baseline levels. Since most DENV pre-exposed volunteers had experienced asymptomatic dengue, it was not possible to correlate the plasmablast response with the time since previous DENV infection.

**Table 2:** Plasmablast percentage in participants allocated to placebo and Butantan-DV vaccinees according to the pre-vaccination DENV serostatus.

Timepoints	DENV-naïve					DENV pre-exposure				
	PlaceboMedian (IQR)	n	VaccineesMedian (IQR)	n	*p-value*	PlaceboMedian (IQR)	n	VaccineesMedian (IQR)	n	*p-value*
Baseline	0.72 (0.33-2.74)	4	1.71 (0.92-2.09)	13	0.245	1.01 (0.63-2.01)	9	0.96 (0.54-1.31)	14	0.249
D6	1.01 (0.45-1.40)	4	0.89 (0.49-1.97)	13	0.623	0.73 (0.45-0.99)	8	0.64 (0.51-2.70)	13	0.804
D11-12	0.65 (0.20-1.68)	4	1.66 (1.06-3.25)	13	0.101	0.71 (0.43-1.38)	8	1.51 (0.98-3.69)	11	0.032
D15	0.35 (0.29-0.93)	3	2.19 (1.19-4.08)	10	0.014	0.68 (0.38-0.86)	9	2.30 (1.40-7.05)	13	<0.001
D21-22	0.39 (0.15-0.78)	4	2.03 (1.35-4.72)	13	0.001	1.08 (0.42-1.32)	8	1.13 (0.86-1.78)	14	0.450

D = days post-vaccination. n = number of samples available for analysis with the Mann-Whitney U test.

Although no difference was observed in the frequency of peak plasmablast levels between DENV-naïve and -exposed vaccinees, the increment in circulating plasmablasts following immunization (measured as the percentage of CD20^-^ CD27^+^ CD38^+^ of CD19^+^ B cells at peak minus baseline) was greater in DENV-exposed participants (median: 1.42, IQR 0.39-5.11) compared to DENV-naïve individuals (median: 0.22, IQR 0.71-1.48; p=0.044, **Figure 4C**). Among DENV-exposed vaccinees, the expansion of the circulating plasmablasts was even higher in those individuals who were seropositive for two or more DENV serotypes prior to vaccination (median: 1.63, IQR 1.08-4.43; p=0.029). Also, in DENV-exposed vaccinees, plasmablast responses transiently peaked at day 15 post-vaccination, reducing subsequently to baseline levels at day 22, suggesting a transient plasmablast expansion. Plasmablast expansion also peaked at day 15 post-vaccination among DENV-naïve vaccinees, but in contrast a high percentage of these cells persisted in the peripheral blood until day 22 (**Figure 4A**).

## Discussion

Vaccine-induced B cell activation has been historically evaluated by methods that measure humoral immunity (28, 29). However, the generation of vaccine-induced Ab titers in the serum often takes from several months to years to fully develop (30, 31). The production of antigen-specific Abs by B-cells is an important component of the immune response and is the basis for most successful vaccination strategies (32). Indeed, Ab-secreting B cells play a central role in the development of dengue immunopathology (12, 18) and their activation is essential for immune protection (33). The measurement of early B cell activation has been described as an initial indicator of immunogenicity (34). Several studies have analyzed the dynamics of Ab-producing B cells in the blood of immunized (16, 23, 35–37) or infected individuals (12, 13, 18, 37–46) by flow cytometry. Plasmablasts can be detected using the surface markers CD19, CD20, CD27 and CD38. Even though we cannot affirm that plasmablast expansion following vaccination is specific to the vaccine antigens/viruses, previous studies have already shown that the circulating plasmablast enhanced following natural infection (12) and TV003 vaccine (47) comprised of mostly DENV-specific IgG secreting plasmablasts. By monitoring the expression of such markers in the peripheral blood, we observed that the timing of plasmablast response after immunization with the Butantan-DV was consistent with other studies in natural DENV infection (12, 13, 18, 38–40) and with one Phase I study of the plasmablast expansion in DENV-naïve individuals who received TV003 (23). It has already been shown that the concentrations of plasmablasts in the blood peaks at days 6-7 after the onset of symptoms for recall responses and somewhat later for primary responses in natural DENV infection (39). The timing of peak plasmablasts induced by the Butantan-DV was not influenced by baseline DENV serostatus.

Here we detected a plasmablast population of more than 30% of the total circulating CD19^+^ B cells in some individuals, similar to that seen in secondary DENV infections (12). As expected, the frequencies of peak plasmablast levels in DENV-naïve and -exposed vaccinees was lower than that seen following natural infection (48), but very similar to the one seen after vaccination with YF-17D, a highly immunogenic and efficacious vaccine (49, 50), and also seen in the Phase I cohort of TV003 (23).

Other candidate vaccines have shown differences in their ability to induce humoral responses following vaccination of flavivirus- and/or DENV-seropositive versus -seronegative individuals. This is an extremely important discussion in regions with endemic circulation of several flavivirus, such as Brazil. In the Dengvaxia trial, DENV-seropositive individuals (PRNT50 ≥1:10 to DENV) had higher nAb titers and a greater proportion of the vaccinees seroconverted following the first two vaccine doses; however, the vaccine had lower efficacy in naïve populations (51, 52). Overall, our data suggest that prior DENV exposure may influence B-cell-mediated responses following vaccination with Butantan-DV, and still allow significant plasmablast expansion and seroconversion in naïve populations as shown here and by others (24). Our PRNT results indicated low nAb titers against DENV-3 in most naïve vaccinees countering previous findings in TV003 recipients (32). One explanation for this in a sense unexpected result may be that the variations on PRNT results among distinct laboratories in response to varied testing conditions (53) that influence the detection of nAb titers to different DENV serotypes (28). This, added to the limited sample size, may have generated divergent data.

Although the median peak percentage of the plasmablast response induced by the Butantan-DV in both DENV-naïve and -exposed groups was comparable, pre-vaccination nAbs appeared to be associated with a greater increase in circulating plasmablasts following immunization compared to baseline plasmablast levels. This was notable among individuals who were seropositive for two or more DENV serotypes prior to vaccination. This finding validates the hypothesis that preexisting DENV immunity can influence the magnitude of the plasmablast response (39) and modulate the Ab response following Butantan-DV, as previously suggested (54).

Our results also indicate that preexisting DENV immunity influences the persistence of plasmablasts in the peripheral blood. In DENV-exposed vaccinees, blood plasmablast concentrations returned to baseline levels 22 days after immunization. In contrast, the plasmablast expansion persisted beyond day 22 in DENV-naïve individuals, as previously shown (23). It is possible that DENV-seronegative volunteers have longer vaccine virus replication, supporting continuous plasmablast expansion. Among vaccinated participants, vaccine-induced viremia occurred most frequently at day 9 post-vaccination for both DENV-naïve or -exposed individuals, but at day 12, detectable viremia was seen mostly among DENV-naïve volunteers (**Supplementary Table 1**). Additionally, memory B-cells are thought to respond and contract more rapidly than their naïve counterparts (47). Thus, it remains possible that the pre-existing responses determined the faster kinetics of B cell expansion and contraction in DENV-exposed volunteers.

## Conclusions

Assessment of plasmablast responses following infection and vaccination has been used as an early biomarker of serologic responses (55). The detection of plasmablast expansion in the blood after recent immunization with the Butantan-DV in naïve and DENV-exposure individuals is, in addition to the traditional serologic markers, another indication of this vaccine’s ability to induce B-cell activation. Therefore, our data provide further confirmation of the immunogenicity of the Butantan-DV.

## Data Availability Statement

The original contributions presented in the study are included in the article/**Supplementary Material**. Further inquiries can be directed to the corresponding author.

## Ethics Statement

The studies involving human participants were reviewed and approved by CAPPesq, Research Projects Ethics Committee, protocols #55308 and #1.213.202 CONEP, protocol #155.880. The patients/participants provided their written informed consent to participate in this study.

## Author Contributions

CS and DM collected, analyzed data and wrote the draft of the manuscript. PC performed experiments and wrote the draft of the manuscript. VA-S performed the statistical analysis. MR performed assays. MT performed experiments and analyzed data. CC and MM wrote sections and revised the manuscript. RG, LF and ZN recruited volunteers and organized the database for the clinical trial. CT and HT processed volunteer samples and organized the database. JK, RP and AP designed the clinical trial. DW and EK contributed to conception of the study and final manuscript revision. All authors contributed to the article and approved the submitted version.

## Funding

This work was supported by funding from the Wallace H. Coulter Center for Translational Research, Miami, FL, the São Paulo Research Foundation (FAPESP) 2015/03933-3, the National Council for Scientific and Technological Development (CNPq), the Brazilian Development Bank (BNDES), and the Intramural Research Program of the National Institute of Allergy and Infectious Diseases (NIAID, NIH).

## Conflict of Interest

JK and RP are former employees of the Butantan Institute. AP is an employee of the Butantan Institute.

The remaining authors declare that the research was conducted in the absence of any commercial or financial relationships that could be construed as a potential conflict of interest.

The reviewer (CP) declared a shared affiliation with the author (MT) to the handling editor at the time of review.

## Publisher’s Note

All claims expressed in this article are solely those of the authors and do not necessarily represent those of their affiliated organizations, or those of the publisher, the editors and the reviewers. Any product that may be evaluated in this article, or claim that may be made by its manufacturer, is not guaranteed or endorsed by the publisher.
