# Supplementary material for: Plasmablast Expansion Following the Tetravalent, Live-Attenuated Dengue Vaccine Butantan-DV in DENV-Naïve and DENV-Exposed Individuals in a Brazilian Cohort
Source: Front Immunol. 2022 Jun 28;13:908398. doi: 10.3389/fimmu.2022.908398 (PMC9274664; doi:10.3389/fimmu.2022.908398)
Supplement: Supplementary file 1 [file DataSheet_1.pdf]

Supplementary Material

Supplementary Figures

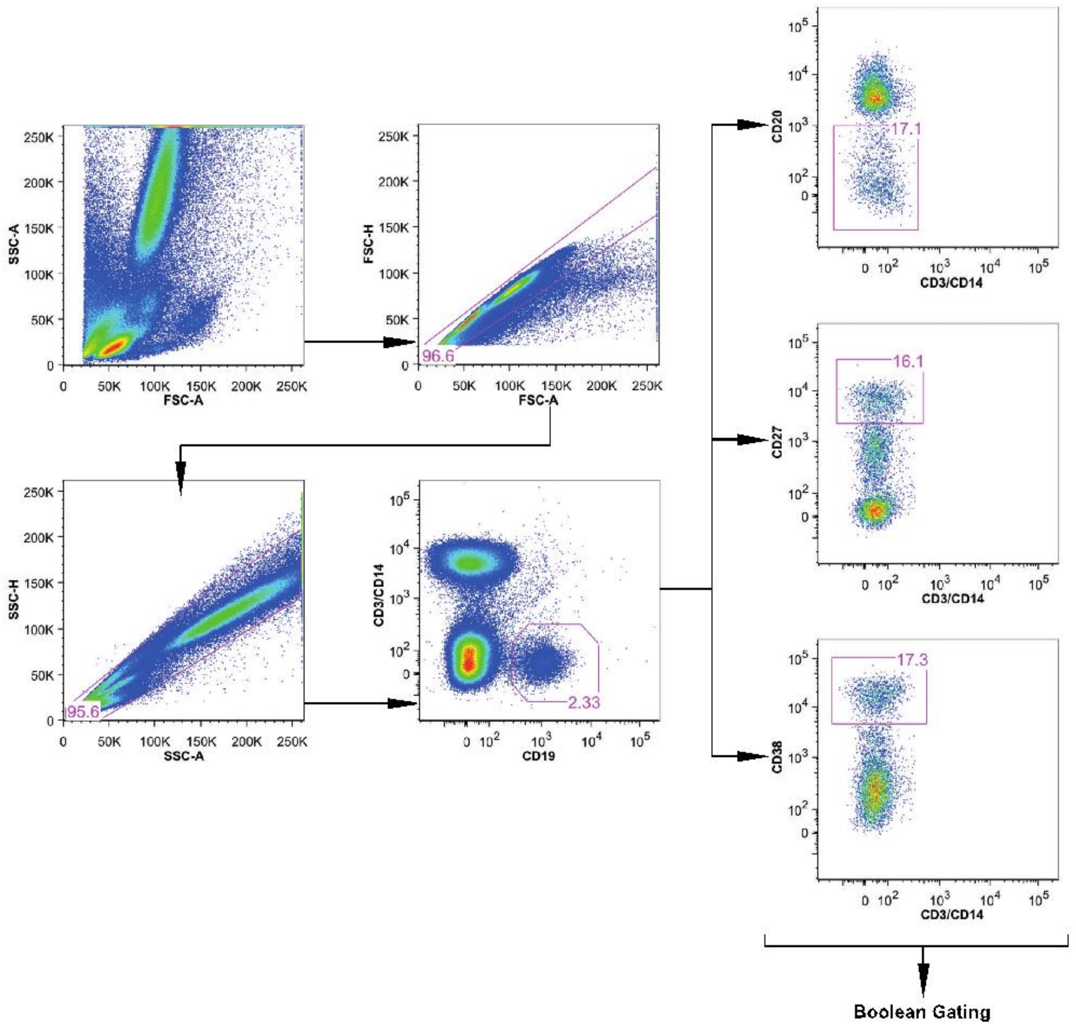

Supplementary Figure 1. Multicolor flow cytometric gating strategy for plasmablasts. The depicted data are from a DENV pre-exposure vaccinee. Following a lymphocyte gate and cell doublet discrimination (FSC-H vs. FSC-A and SSC-H vs. SSC-A), cells were further gated for live CD3- CD14- CD19+ cells and then they were analyzed for surface expression of CD20, CD27 and CD38. The expression profile of each marker for identifying plasmablast cells was boxed. Boolean gating was performed to generate frequencies of the circulating plasmablasts in the live CD3- CD14- CD19+ cells at each time point.

Supplementary Table 1. Butantan-DV recipients with detectable viremia from vaccine viruses after immunization, stratified by previous exposure to DENV.

	Days after immunization					
	**3**	**6**	**9**	**12**	**15**	**21**
**DENV-naive** (n=14)	0 (0%)	5 (35,7%)	8 (57,1%)	5 (35,7%)	0 (0%)	0 (0%)
**DENV pre-exposed** (n=14)	1 (7,1%)	2 (14,3%)	7 (50%)	3 (21,4%)	0 (0%)	0 (0%)
**Total** (n=27)	1 (3,7%)	7 (25,9%)	15 (55,6%)	8 (29,6%)	0 (0%)	0 (0%)
